# Elasmobranch bycatch in the Italian Adriatic pelagic trawl fishery

**DOI:** 10.1371/journal.pone.0191647

**Published:** 2018-01-29

**Authors:** Sara Bonanomi, Jacopo Pulcinella, Caterina Maria Fortuna, Fabrizio Moro, Antonello Sala

**Affiliations:** 1 Italian National Research Council (CNR), Institute of Marine Sciences (ISMAR), Ancona, Italy; 2 Italian National Institute for Environmental Protection and Research (ISPRA), Rome, Italy; University of Padova, ITALY

## Abstract

Elasmobranchs are among the most threatened long-lived marine species worldwide, and incidental capture is a major source of mortality. The northern central Adriatic Sea, though one of the most overfished basins of the Mediterranean Sea, supports a very valuable marine biodiversity, including elasmobranchs. This study assesses the impact of the northern central Adriatic pelagic trawl fishery on common smooth-hound (*Mustelus mustelus*), spiny dogfish (*Squalus acanthias*), common eagle ray (*Myliobatis aquila*), and pelagic stingray (*Pteroplatytrygon violacea*) by examining incidental catches recorded between 2006 and 2015. The distribution of bycatch events was evaluated using geo-referenced data. Generalized Linear Models were computed to standardize the catch of the four species and to predict the relative abundance of bycatch events. Data analysis shows that most bycatch events involving all four species occurred in the northern Adriatic Sea. The models predicted significant, distinct temporal patterns of standardized catches in line with previous investigations. Water depth, season, and fishing region were the best predictors to explain bycatch events. The present data suggest that the northern Adriatic may be an important nursery area for several elasmobranchs. They also highlight the urgent need for a better understanding of the interactions between elasmobranchs and fisheries to develop and apply suitable, ad hoc management measures.

## Introduction

The unintentional capture of non-target species, or bycatch, occurring during fishing operations [1, 2] is still a major source of anthropogenic mortality hampering the survival of species of conservation concern worldwide [3, 4]. Several elasmobranch species are held to be particularly vulnerable to the effects of bycatch due to their long lifespan, late age at maturity, large size at birth, and low reproductive rates [5, 6]. Such biological characteristics limit the ability of populations to recover from heavy fishing pressure [7], thus contributing to their decline [4, 8]. According to the International Union for the Conservation of Nature (IUCN) Red List, approximately 24% of cartilaginous fish belong to a threatened category [9]. Recent estimates by the United Nations Food and Agriculture Organization (FAO) indicate that about 766,000 tons of chondrichthyes, mainly sharks and batoids, were caught in 2011 [10]. A substantial portion of these catches was unintentional. Major threats to elasmobranchs from fisheries activities include entanglement, hooking, or trapping by fishing gears that are usually intended to catch valuable commercial species. Although a number of studies have investigated the impact of fishing gears on long-lived marine species of conservation concern, like cartilaginous fish (for a review see [5, 11–13]), further research is needed to establish how mortality due to interactions with fisheries varies by species and gear type.

The northern central Adriatic Sea is the most heavily impacted basin in the Mediterranean Sea due to a variety of sources of anthropogenic pressure, mainly intense fishing activities, large urbanized and industrialized areas, and environmental pollution [14–17]. Yet, the area supports a rich and valuable marine biodiversity including long-lived marine species like elasmobranchs. Interactions between these species and fisheries are therefore unavoidable. Indeed, a dramatic decline of large and small elasmobranchs, as inferred from landing and abundance data, has recently been reported in the northern Adriatic [18, 19], where landings have traditionally been dominated by *Mustelus spp*. and *Squalus spp*. mainly taken as bycatch [19, 20]. However, data on the extent of bycatch of these and other cartilaginous fish in the Adriatic are still limited.

Since 2006, an extensive monitoring programme of accidental catches of long-lived species like cetaceans, sea turtles, and elasmobranchs by Italian pelagic trawlers has been conducted in the northern central Adriatic Sea [21–23]. The information collected in its framework provides a unique opportunity to assess the operational details of capture events and the abundance trends of species over time. The present study evaluates the impact of pelagic trawling on four elasmobranch species—common smooth-hound (*Mustelus mustelus*), common eagle ray (*Myliobatis aquila*), spiny dogfish (*Squalus acanthias*), and pelagic stingray (*Pteroplatytrygon violacea*)—in the northern central Adriatic Sea. Those species were selected based on the limited or absence of information on their biology and incidental catches in the study area. The objectives are to (i) assess the distribution of bycatch events involving the four elasmobranchs; (ii) examine their relative abundance between 2006 and 2015 using Catch-Per-Unit-Effort (CPUE) time series; and (iii) predict the spatial and seasonal scale of bycatch events in this area. The analytical approach used in this study include a zero-inflated model and selected a zero-inflated negative binomial model (ZINB) for CPUE standardization. This is one the most useful method to deal with several zero observations [24]. The information gained from this study can contribute to improve fisheries management and to develop measures aimed at reducing bycatch events of a variety of cartilaginous fish species.

## Materials and methods

### Ethics statement

In European waters, elasmobranchs are generally considered commercial species, except for the basking shark (*Cetorinhus maximus*) and the white shark (*Carcharodon carcharias*), which are fully protected from all fisheries (Regulations (EC) 40/2007, 41/2007). Other species such as the bluntnose sixgill shark (*Hexancus griseus*), thresher sharks (*Alopiidae spp*.*)*, whale shark (*Rhincodon typus*), requiem sharks (*Carcharhinidae spp*.), hammerhead, bonnethead and scoophead sharks (*Sphyrnidae spp*.) and mackerel sharks (*Isuridae* and *Lamnida*) are protected from driftnetting (Regulation (EC) 894/97 amended). Under Regulation (EC) 43/2009 quotas are set for other species of elasmobranchs in some northern European fishing areas. The study was entirely based on incidental catches of non-protected elasmobranch species recorded by qualified observers on board Italian pelagic trawlers in the Adriatic Sea between 45°2’9.6” and 42°30’. The data collection was conducted under permit issued by the Italian Ministry of Agriculture and Forestry, Fishery and Aquaculture directorate in compliance with the Italian obligations to the Council Regulation (EC) 812/2004. No other authorization or ethics board approval was required to conduct the study.

### Study area

**Fig 1:**
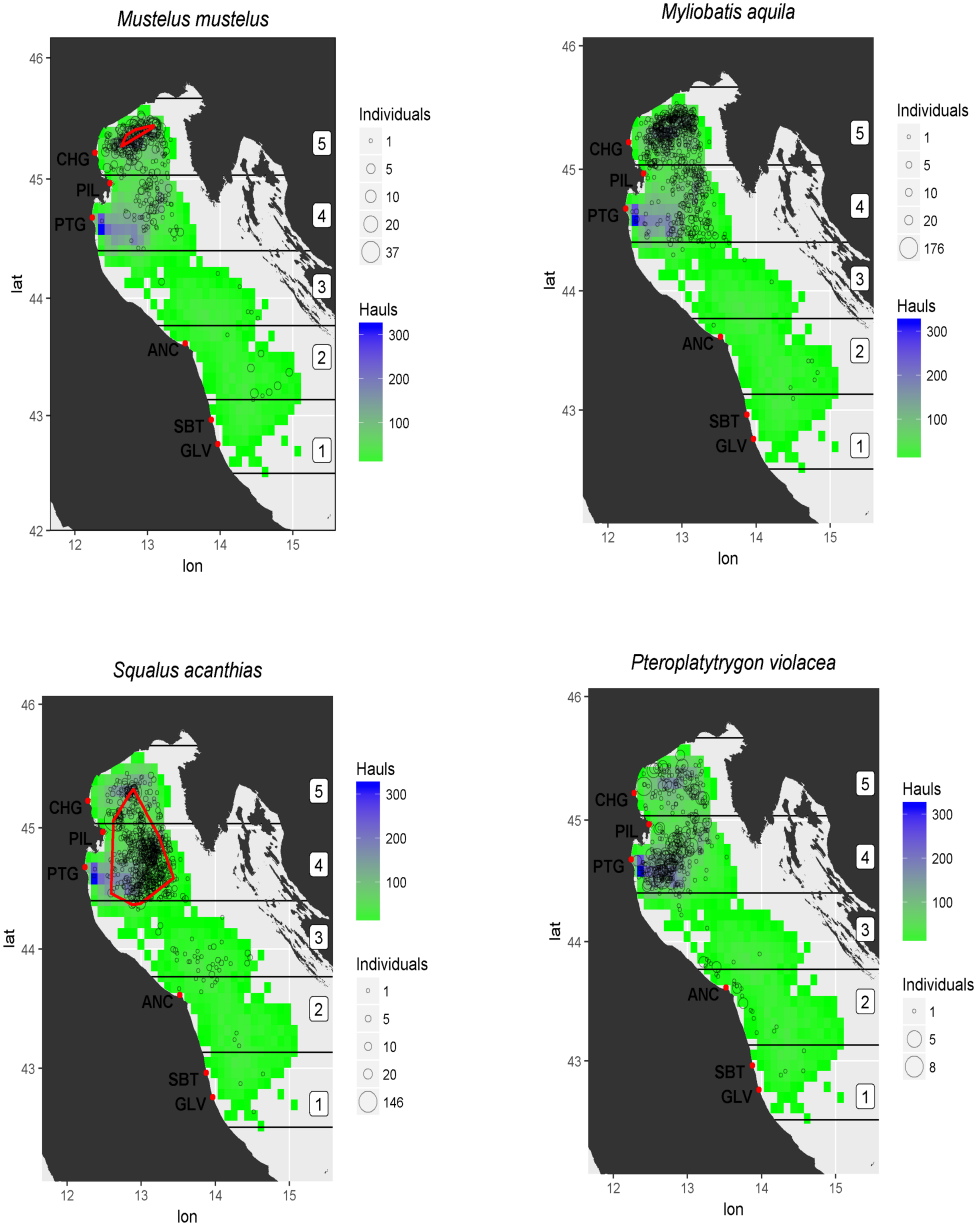
Geographic distribution of hauls and bycatch events of *Mustelus mustelus* (a), *Squalus acanthias*, (b), *Myliobatis aquila* (c), and *Pteroplatytrygon violacea* (d), collected between 2006–2015. The number of hauls is illustrated with a colour gradient from green (0–100 hauls) to purple-blue (200–300 hauls). The size of circles indicates the magnitude of bycatch events. Nursery areas of *M*.*mustelus* and *S*. *acanthias* are reported in red. The number in the square boxes on the right side of the maps indicate the fishing regions in which the study area was divided.

The Italian pelagic trawl fishery is mainly based in the northern and central basin of the Adriatic basin, Geographical Sub-Area (GSA) 17 (Fig 1). The basin is characterized by shallow waters, with an average depth of 35 m. The strong influence of the Po river plumes results in low salinity, low water temperature, and high productivity [25–27]. GSA 17 covers the entire northern and central Adriatic Sea as far as the Gargano Promontory in Italy and the city of Kotor in Montenegro.

### Pelagic trawling

Pelagic (or midwater) trawling in the northern Adriatic developed in the 1950s [28], targeting mostly small pelagic fish such as anchovy (*Engraulis encrasicolus*) and sardine (*Sardine pilchardus*). About 80 Italian semi-pelagic trawlers currently operate in shallow waters (less than 100 m) during the day, when small pelagic schools swim close to the bottom [29]. Midwater trawling is carried out by two paired vessels having similar size and engine power (average engine power is 440 HP, average length overall LOA is 25 m and average vessel size is 90 GT). The midwater trawl net consists of a cone- or pyramid-shaped body whose size depends on engine power, available towing pull, and vessel size. Its length is usually 60–70 m from the wings to the codend and the rigging is designed to fish in midwater [30, 31]. The horizontal opening of the net is provided by the distance between the two vessels during towing, whereas its vertical opening is ensured by floats on the headline and weights on the groundline. The minimum mesh size of the codend of the “volante” trawl, where at least 80% of the catch in live weight after sorting is made up of sardine and anchovy, is 20 mm (EC Regulation 1967/2006).

### Data collection

Between 2006 and 2015, qualified observers on board 57 pelagic trawlers (see Table 1) operating in the northern central Adriatic Sea monitored all fishing operations and collected bycatch data of protected species (e.g., cetaceans) and species of conservation concern (e.g., elasmobranchs). For each haul, they recorded operational parameters including haul duration, time of net setting and hauling, and trawling speed (nm); environmental variables like geographical coordinates (latitude and longitude in WGS84), water depth (m), and date.In this study, bycatch data of four elasmobranch species—common smooth hound (*Mustelus mustelus*), common eagle ray (*Myliobatis aquila*), spiny dogfish (*Squalus acanthias*), and pelagic stingray (*Pteroplatytrygon violacea*)—were considered (see Table 1).

**Table 1:** Average of Italian pelagic trawlers monitored between 2006 and 2015 in five different fishing regions (1–5) in the northern central Adriatic Sea. Average vessel characteristics (LOA, GT and kW) are also indicated.

Fishing region ^a^	Year	N. of vessels	LOA ^b^	GT ^c^	kW ^d^
1	2006	2	26.35	83	625
	2007	2	26.35	83	625
	2008	3	26.18	85	655
	2009	2	28.85	144	496
	2010	7	27.21	104	562
	2011	1	28.70	157	515
	2012	2	27.90	143.5	419
	2013	0	0	0	0
	2014	2	28.45	123.5	515
	2015	2	0	0	0
2	2006	2	26.35	83	624.95
	2007	3	26.18	85	655.3
	2008	3	26.18	85	655.3
	2009	3	27.95	121.6	594.3
	2010	17	26.50	109.8	443
	2011	5	27.66	119.8	490.2
	2012	11	27.14	119	445
	2013	6	27.42	116	540
	2014	2	28.45	123.5	515
	2015	5	16.39	68.2	380.2
3	2006	0	0	0	0
	2007	0	0	0	0
	2008	4	21.75	46.12	373
	2009	8	25.35	92	501
	2010	18	26.07	109	472
	2011	8	26.76	115	518
	2012	13	26.34	113	493.4
	2013	8	25.56	98	551.25
	2014	0	0	0	0
	2015	8	26	94	569
4	2006	4	26	57.29	485.5
	2007	6	23.70	55.42	428.9
	2008	9	23.93	67.59	432.0
	2009	6	23.82	79.77	490.2
	2010	7	23.67	78.69	469.5
	2011	7	23.46	88.59	408.4
	2012	9	24.16	96.58	464.6
	2013	9	24.91	95.16	488.2
	2014	4	20.58	52.03	355.0
	2015	8	25.36	93.62	494.5
5	2006	2	28.42	57.85	576.5
	2007	3	25.21	65.44	439.8
	2008	5	24.88	73.35	485.5
	2009	0	0	0	0
	2010	4	24.66	96.69	490.5
	2011	5	23.75	96.66	401.6
	2012	7	24.54	104.6	473.45
	2013	9	25.62	94.7	0
	2014	0	0	0	482.7
	2015	5	26.01	99.15	514.1

^a^ The study area was divided in five fishing regions according to the latitudinal range of fishing operations: southern, central-southern, central, central-northern and northern (#1–5).

^b^ LOA: overall length.

^c^ GT: total tonnage.

^d^ kW: engine power.

### CPUE standardization

The most common method to summarize fishery data is to use Catch-Per-Unit-Effort (i.e. CPUE) as a measure of relative abundance. However, since the catch rate is influenced by multiple factors, raw CPUE data cannot be considered as being proportional to abundance, thus raising interpretation problems [32–34]. To overcome this difficulty, CPUE data are generally standardized by removing the effect of factors that influence the catch rate [24]. In the case of non-target species, like elasmobranchs in northern central Adriatic pelagic/midwater trawl fisheries, catch rate standardization requires addressing several questions. Since such species are generally caught less frequently than target species, catch data may also be characterized by several zero observations (no catch). Classic count models, like Poisson or negative binomial distribution, are unable to deal with an excess of zeros, resulting in inference problems [35]. Zero-inflated General Linear Models (GLMs) are considered appropriate to standardize bycatch species CPUE data [24, 36]. These models consist of two-components that treat zeros and counts as two different source of information [35]. One component is a binomial model, which measures the probability to observe an extra zero, called also “false zeros”, which in case of catch data are attributable to survey error, misreporting or reporting error [35]. A second component is a non-negative count distribution, usually a Poisson or a negative binomial model, used to modelling count process that could be a positive count or equal to zero (in this case a “true zero” catch). Depending on the type of distribution used in the count component, these models are referred as Zero-Inflated Poisson (ZIP) and Zero-Inflate Negative Binomial (ZINB).

In this study, a ZINB model was fitted for each species analysed after a variable selection procedure aimed to identify the most appropriate set of covariate to use in the models. The explanatory variables to be included in the final ZINB models were identified by backward elimination. Given that different covariates could be included in the binomial and in the count model, the best set of explanatory variables was selected starting from the model containing the full set in both components. The explanatory variables are described below. Temporal trend of CPUE was assessed considering 10 fishing years (2006–2015) and 4 calendar quarters (1Q-4Q). The regional effect was measured, due to its important role in the spatial resolution of bycatch events. It was assessed with a categorical variable having 5 levels which correspond to 5 fishing regions obtained dividing the study area according to the latitudinal range of fishing operations: southern, central-southern, central, central-northern and northern (#1–5).

Common smooth-hound and spiny dogfish may form large schools of adult individuals during the mating season as well as aggregations of immature individuals in their nursey areas [36, 37]. For these two species, the mating and nursery effects were tested, because they were expected to influence the magnitude of bycatch events. Information on the mating period was obtained from the best available literature [37]. The effect of mating behaviour was considered as a factor variable, with two levels distinguishing observations made during breeding and in other periods. The nursery areas were identified following an empirical procedure based on the identification of the minimal area in which were recorded large bycatch events with immature individuals. As a first step, for both common smooth hound and spiny dogfish, length at first maturity reported in [36] was considered as the threshold to identify immature individuals (see S1 Table). Considering these values, for each haul only bycatch events with at least 50% of immature individuals caught were selected. Following this, aggregations with more than 18 and 8 individuals were selected respectively for common smooth hounds and spiny dog fish. These thresholds represented the 95th quantile of the distribution of catches of immature individuals. Finally, for both species the minimal area containing the selected events was computed and considered as a potential nursery area. Similarly to mating effect, the effect of the nursery area was tested by distinguishing observations made in the nursery area from those made outside it.

The measures used to describe the habitat preferences of the four species were depth of sea bottom (expressed as the logarithm of mean depth recorded during a haul) and sea surface temperature (SST). Daily SST was obtained from the global ocean physics analysis and forecast system (GLOBAL_ANALYSIS_FORECAST_PHY_001_024), distributed by the Copernicus Marine Environment Monitoring Service (CMEMS; http://marine.copernicus.eu/). Since hauls were longer in the southern regions than in the northern regions, the fishing effort was included in the model as an offset and computed as the logarithm of haul duration expressed in hours. The offset was included only in the count component of the ZINB model.

Models evaluated during backward elimination were assessed by the Akaike Information Criterion (AIC) and the model with the lowest value was used. The significance of the variables used in the final models was assessed by likelihood ratio test by dropping one variable at time. For each variable dropped, the difference in units of AIC was estimated between the starting model and the model without the dropped variable [38].

The standardized CPUEs were compared to mean nominal annual CPUE (*CPUEnom*_*y*_) which was computed as the product of average haul duration (${\overset{-}{D}}_{y}$) and the mean number of elasmobranch individuals caught in each haul (${\overset{-}{I}}_{y}$):

$${CPUEnom}_{y}\;=\;{\overset{-}{D}}_{y}*{\overset{-}{I}}_{y}$$

The effects of mating behaviour and nursery area on the catch rate of common smooth-hound and spiny dogfish were explored in terms of differences in mean CPUE using a pairwise comparison of estimated values. Finally, to analyse the seasonal and spatial pattern of the catch rate, fitted effect coefficients of the standardized CPUE were used to predict the bycatch of the four species in terms of number of individuals found in each haul by region and quarter along depth gradient. Since bycatch events in the southern region were few for all species, the area was excluded from data analysis due to lack of convergence occurring during model fitting. For the same reason all combinations of regions and seasons for which the empirical bycatch frequency was < 0.01 were also excluded.

### Computation

All GLM computations were performed using R Version 3.3.1 for Linux [39]. The ZINB models were computed with the “pscl” library. Catch data dispersion was tested using the dispersion test implemented in the “AER” library [40]. Pairwise comparisons were performed with the “stats” library. The significance criterion for statistical tests was P < 0.05.

## Results

### Empirical patterns of monitored fishing activity and bycatch events

A summary statistic of the data is reported in Table 2. A total of 12,585 successful hauls were recorded in 3,160 monitored fishing trips. Between 2006 and 2015, the average monitoring effort was about 1,258 hauls per year. Specifically, 52% and 27% of monitored hauls were performed in the central-northern and northern fishing regions (#4 and 5), respectively, 8% in the central and central-southern fishing regions (#2 and 3), and 3% in the southern fishing region (#1). The geographical distribution of bycatch events of the four elasmobranch species considered is illustrated in Fig 1. The 1,932 hauls involving positive bycatch events (at least one individual caught) comprised 5,436 individuals. Most events were in regions #4 and 5, which accounted for 94% of total bycatch events. Based on these data, the empirical pattern of bycatch frequency (number of hauls with at least one individual caught divided by the total number of hauls monitored) was fairly low, with an average value of about 0.04. In particular, 2,169 spiny dogfish were captured (average frequency, 0.061), followed by 833 common smooth-hound (average frequency, 0.027), 1,880 common eagle ray (average frequency, 0.054), and 555 pelagic stingray (average frequency, 0.033) individuals. These data are reported in Table 2. Most spiny dogfish (70%) and smooth-hound (79%) individuals were retained on board, whereas most common eagle ray (79%) and pelagic stingray (90%) individuals were released alive.

**Table 2:** Summary table reporting the number of hauls (with zero and positive catches) recorded during the observing programme conducted between 2006 and 2015. Bycatch frequency, total number of individuals caught, and mean CPUE for each of the four species, five fishing regions (1–5) with latitudinal range and medium depth, and calendar quarters (1Q-4Q) are also showed.

***a) Mustelus mustelus***																					
**Year**		**1** **(42.7–43.4 91 m)**				**2** **(43.5–44.0 71 m)**				**3** **(44.1–44.6 58 m)**				**4** **(44.7–45.2 31)**				**5** **(45.3–45.8 39)**			
		**Q1**	**Q2**	**Q3**	**Q4**	**Q1**	**Q2**	**Q3**	**Q4**	**Q1**	**Q2**	**Q3**	**Q4**	**Q1**	**Q2**	**Q3**	**Q4**	**Q1**	**Q2**	**Q3**	**Q4**
**2006**	**Hauls 0 catches**				31				46							74				212	166
	**Hauls with catches**				0				0							0				21	7
	**Bycatch frequency**				0				0							0				0.09	0.04
	**Individuals caught**				0				0							0				54	11
	**Mean CPUE**				0				0							0				0.24	0.06
**2007**	**Hauls 0 catches**	31				51								88		117					134
	**Hauls with catches**	0				0								0		2					7
	**Bycatch frequency**	0				0								0		0.02					0.05
	**Individuals caught**	0				0								0		2					8
	**Mean CPUE**	0				0								0		0.02					0.06
**2008**	**Hauls 0 catches**		59			56	31		41	51				358	298	156		23	212	176	119
	**Hauls with catches**		0			0	0		0	0				1	0	0		0	7	7	1
	**Bycatch frequency**		0			0	0		0	0				0.003	0	0		0	0.03	0.04	0.008
	**Individuals caught**		0			0	0		0	0				1	0	0		0	12	14	1
	**Mean CPUE**		0			0	0		0	0				0.004	0	0		0	0.05	0.07	0.005
**2009**	**Hauls 0 catches**				37				25			35	30	29	42	152					
	**Hauls with catches**				0				0			0	0	0	0	9					
	**Bycatch frequency**				0				0			0	0	0	0	0.06					
	**Individuals caught**				0				0			0	0	0	0	13					
	**Mean CPUE**				0				0			0	0	0	0	0.08					
**2010**	**Hauls 0 catches**	56	59		25	55	68		76	90	58		126	309	267	203	200	73	186	125	64
	**Hauls with catches**	0	0		0	1	0		0	0	0		1	0	3	3	3	0	22	0	1
	**Bycatch frequency**	0	0		0	0.02	0		0	0	0		0.008	0	0.01	0.01	0.01	0	0.11	0	0.015
	**Individuals caught**	0	0		0	1	0		0	0	0		2	0	4	6	6	0	55	0	1
	**Mean CPUE**	0	0		0	0.02	0		0	0	0		0.014	0	0.02	0.03	0.02	0	0.27	0	0.011
**2011**	**Hauls 0 catches**		25	30	26			38	160				115	101	93	70	481	34	248	69	46
	**Hauls with catches**		0	0	0			0	0				0	1	0	0	5	0	24	1	0
	**Bycatch frequency**		0	0	0			0	0				0	0.009	0	0	0.01	0	0.09	0.01	0
	**Individuals caught**		0	0	0			0	0				0	1	0	0	5	0	47	6	0
	**Mean CPUE**		0	0	0			0	0				0	0.009	0	0	0.008	0	0.18	0.07	0
**2012**	**Hauls 0 catches**		49			77	102			98	88	0	26	263	224	38	398	0	210	129	136
	**Hauls with catches**		0			6	0			0	0	0	0	3	0	1	8	0	21	9	7
	**Bycatch frequency**		0			0.07	0			0	0	0	0	0.01	0	0.02	0.02	0	0.09	0.06	0.049
	**Individuals caught**		0			14	0			0	0	0	0	7	0	1	9	0	71	26	16
	**Mean CPUE**		0			150	0			0	0	0	0	0.02	0	0.02	0.02	0	0.27	0.17	0.09
**2013**	**Hauls 0 catches**						28	55		80	84	34		287	251	26	122	24	265	99	79
	**Hauls with catches**						0	0		0	0	0		2	0	1	10	0	56	13	1
	**Bycatch frequency**						0	0		0	0	0		0.007	0	0.04	0.07	0	0.17	0.12	0.012
	**Individuals caught**						0	0		0	0	0		4	0	1	18	0	246	35	1
	**Mean CPUE**						0	0		0	0	0		0.009	0	0.04	0.15	0	0.74	0.29	0.019
**2014**	**Hauls 0 catches**	30				27								156	59						
	**Hauls with catches**	0				0								0	0						
	**Bycatch frequency**	0				0								0	0						
	**Individuals caught**	0				0								0	0						
	**Mean CPUE**	0				0								0	0						
**2015**	**Hauls 0 catches**		26				42	26	41		42	46	48	88	283	106	333	22	170	108	63
	**Hauls with catches**		0				2	0	0		0	0	0	1	1	0	10	1	33	9	1
	**Bycatch frequency**		0				0.04	0	0		0	0	0	0.01	0.003	0	0.03	0.04	0.16	0.07	0.015
	**Individuals caught**		0				5	0	0		0	0	0	1	1	0	11	1	58	22	1
	**Mean CPUE**		0				0.08	0	0		0	0	0	0.01	0.004	0	0.03	0.03	0.28	0.20	0.013
***b) Squalus acanthias***																					
**Year**		**1** **(42.7–43.4 91 m)**				**2** **(43.5–44.0 71 m)**				**3** **(44.1–44.6 58 m)**				**4** **(44.7–45.2 31)**				**5** **(45.3–45.8 39)**			
		**Q1**	**Q2**	**Q3**	**Q4**	**Q1**	**Q2**	**Q3**	**Q4**	**Q1**	**Q2**	**Q3**	**Q4**	**Q1**	**Q2**	**Q3**	**Q4**	**Q1**	**Q2**	**Q3**	**Q4**
**2006**	**Hauls 0 catches**				31				46							74	122			232	173
	**Hauls with catches**				0				0							0	6			1	0
	**Bycatch frequency**				0				0							0	0.05			0.004	0
	**Individuals caught**				0				0							0	7			3	0
	**Mean CPUE**				0				0							0	0.06			0.02	0
**2007**	**Hauls 0 catches**	31				51								84		116	355				140
	**Hauls with catches**	0				0								4		3	23				1
	**Bycatch frequency**	0				0								0.04		0.02	0.06				0.007
	**Individuals caught**	0				0								15		10	58				1
	**Mean CPUE**	0				0								0.17		0.09	0.13				0.005
**2008**	**Hauls 0 catches**		58			56	31		41	51				306	291	147	71	22	209	181	119
	**Hauls with catches**		1			0	0		0	0				53	7	9	5	1	10	2	1
	**Bycatch frequency**		0.02			0	0		0	0				0.15	0.02	0.06	0.06	0.04	0.04	0.01	0.008
	**Individuals caught**		1			0	0		0	0				102	10	75	9	1	33	51	1
	**Mean CPUE**		0.02			0	0		0	0				0.28	0.03	0.50	0.12	0.06	0.21	0.23	0.007
**2009**	**Hauls 0 catches**				37				25			33	30	29	42	140	223				
	**Hauls with catches**				0				0			2	0	0	0	21	33				
	**Bycatch frequency**				0				0			0.06	0	0	0	0.13	0.13				
	**Individuals caught**				0				0			36	0	0	0	48	106				
	**Mean CPUE**				0				0			1.20	0	0	0	0.27	0.40				
**2010**	**Hauls 0 catches**	56	37		25	56	67		76	90	56		120	262	263	181	179	69	198	123	64
	**Hauls with catches**	0	0		0	0	1		0	0	2		7	47	7	25	24	4	10	2	1
	**Bycatch frequency**	0	0		0	0	0.01		0	0	0.03		0.05	0.15	0.02	0.12	0.12	0.05	0.05	0.02	0.01
	**Individuals caught**	0	0		0	0	1		0	0	3		13	136	19	76	78	4	17	2	3
	**Mean CPUE**	0	0		0	0	0.02		0	0	0.05		0.10	0.41	0.05	0.33	0.36	0.05	0.07	0.02	0.05
**2011**	**Hauls 0 catches**		25	28	26			36	158				101	62	85	61	425	29	269	70	43
	**Hauls with catches**		0	2	0			2	2				14	40	8	9	61	5	3	0	3
	**Bycatch frequency**		0	0.07	0			0.05	0.01				0.12	0.40	0.09	0.13	0.12	0.15	0.01	0	0.06
	**Individuals caught**		0	2	0			2	2				25	100	10	14	123	10	4	0	5
	**Mean CPUE**		0	0.06	0			0.05	0.01				0.24	1.06	0.09	0.20	0.25	0.30	0.02	0	0.12
**2012**	**Hauls 0 catches**		49			83	101			98	89		26	230	213	37	367		218	126	135
	**Hauls with catches**		0			0	1			0	0		0	36	11	2	39		13	12	8
	**Bycatch frequency**		0			0	0.009			0	0		0	0.13	0.05	0.05	0.09		0.06	0.09	0.05
	**Individuals caught**		0			0	1			0	0		0	84	15	16	108		26	173	19
	**Mean CPUE**		0			0	0.006			0	0		0	0.27	0.06	0.49	0.24		0.12	1.17	0.17
**2013**	**Hauls 0 catches**						28	51		80	85	29		245	240	27	106	24	301	103	70
	**Hauls with catches**						0	4		0	0	5		44	11	0	26	0	20	9	10
	**Bycatch frequency**						0	0.07		0	0	0.15		0.15	0.04	0	0.19	0	0.06	0.08	0.12
	**Individuals caught**						0	4		0	0	5		112	26	0	48	0	165	22	28
	**Mean CPUE**						0	0.07		0	0	0.15		0.33	0.10	0	0.42	0	0.41	0.17	0.43
**2014**	**Hauls 0 catches**	30				27								151	58						
	**Hauls with catches**	0				0								5	1						
	**Bycatch frequency**	0				0								0.03	0.02						
	**Individuals caught**	0				0								14	1						
	**Mean CPUE**	0				0								0.13	0.01						
**2015**	**Hauls 0 catches**		23				44	26	41			45	46	84	272	105	330	22	189	115	58
	**Hauls with catches**		0				0	0	0			1	2	5	12	1	13	1	14	2	6
	**Bycatch frequency**		0				0	0	0			0.02	0.04	0.05	0.04	0.009	0.04	0.04	0.07	0.02	0.09
	**Individuals caught**		0				0	0	0			1	3	7	22	2	14	1	20	2	7
	**Mean CPUE**		0				0	0	0			0.01	0.05	0.08	0.05	0.02	0.04	0.08	0.10	0.02	0.11
***c) Myliobatis aquila***																					
**Year**		**1** **(42.7–43.4 91 m)**				**2** **(43.5–44.0 71 m)**				**3** **(44.1–44.6 58 m)**				**4** **(44.7–45.2 31)**				**5** **(45.3–45.8 39)**			
		**Q1**	**Q2**	**Q3**	**Q4**	**Q1**	**Q2**	**Q3**	**Q4**	**Q1**	**Q2**	**Q3**	**Q4**	**Q1**	**Q2**	**Q3**	**Q4**	**Q1**	**Q2**	**Q3**	**Q4**
**2006**	**Hauls 0 catches**				31				46							72	121			186	161
	**Hauls with catches**				0				0							2	7			47	12
	**Bycatch frequency**				0				0							0.03	0.05			0.20	0.07
	**Individuals caught**				0				0							6	12			83	14
	**Mean CPUE**				0				0							0.09	0.10			0.35	0.07
**2007**	**Hauls 0 catches**	31				50								88		113	371				135
	**Hauls with catches**	0				1								0		6	7				6
	**Bycatch frequency**	0				0.02								0		0.05	0.02				0.04
	**Individuals caught**	0				1								0		10	7				8
	**Mean CPUE**	0				0.03								0		0.07	0.02				0.06
**2008**	**Hauls 0 catches**		59			56	31		41	51				358	292	151	72	23	212	155	120
	**Hauls with catches**		0			0	0		0	0				1	6	5	4	0	0	28	0
	**Bycatch frequency**		0			0	0		0	0				0.003	0.02	0.03	0.05	0	0	0.15	0
	**Individuals caught**		0			0	0		0	0				1	10	11	8	0	0	76	0
	**Mean CPUE**		0			0	0		0	0				0.004	0.04	0.08	0.11	0	0	0.40	0
**2009**	**Hauls 0 catches**				37				25			35	30	29	41	153	252				
	**Hauls with catches**				0				0			0	0	0	1	8	4				
	**Bycatch frequency**				0				0			0	0	0	0.02	0.05	0.02				
	**Individuals caught**				0				0			0	0	0	1	16	4				
	**Mean CPUE**				0				0			0	0	0	0.02	0.08	0.01				
**2010**	**Hauls 0 catches**	56	37		25	56	68		74	89	58		127	304	269	193	192	73	172	112	63
	**Hauls with catches**	0	0		0	0	0		2	1	0		0	5	1	13	11	0	36	13	2
	**Bycatch frequency**	0	0		0	0	0		0.03	0.01	0		0	0.02	0.003	0.06	0.05	0	0.17	0.10	0.03
	**Individuals caught**	0	0		0	0	0		2	1	0		0	6	1	23	21	0	220	41	2
	**Mean CPUE**	0	0		0	0	0		0.02	0.04	0		0	0.02	0.003	0.10	0.10	0	1.18	0.31	0.02
**2011**	**Hauls 0 catches**		25	30	25			38	160				115	87	88	67	440	34	246	62	38
	**Hauls with catches**		0	0	1			0	0				0	15	5	3	46	0	26	8	8
	**Bycatch frequency**		0	0	0.04			0	0				0	0.15	0.05	0.04	0.09	0	0.09	0.11	0.17
	**Individuals caught**		0	0	1			0	0				0	40	9	3	86	0	110	29	14
	**Mean CPUE**		0	0	0.03			0	0				0	0.42	0.12	0.06	0.18	0	0.37	0.42	0.31
**2012**	**Hauls 0 catches**		49			81	102			97	91		23	255	223	35	381		180	102	119
	**Hauls with catches**		0			2	0			1	1		3	11	1	4	25		51	36	24
	**Bycatch frequency**		0			0.02	0			0.01	0.011		0.11	0.04	0.004	0.10	0.06		0.22	0.26	0.17
	**Individuals caught**		0			2	0			1	1		4	26	1	28	202		199	137	37
	**Mean CPUE**		0			0.02	0			0.01	0.008		0.21	0.07	0.003	0.55	0.37		0.88	1.27	0.26
**2013**	**Hauls 0 catches**						28	54		80	84	34		284	247	27	122	24	265	79	68
	**Hauls with catches**						0	1		0	1	0		5	4	0	10	0	56	33	12
	**Bycatch frequency**						0	0.02		0	0.02	0		0.02	0.02	0	0.07	0	0.17	0.29	0.15
	**Individuals caught**						0	1		0	1	0		5	28	0	10	0	145	60	21
	**Mean CPUE**						0	0.02		0	0.009	0		0.02	0.10	0	0.07	0	0.42	0.57	0.30
**2014**	**Hauls 0 catches**	30				27								156	59						
	**Hauls with catches**	0				0								0	0						
	**Bycatch frequency**	0				0								0	0						
	**Individuals caught**	0				0								0	0						
	**Mean CPUE**	0				0								0	0						
**2015**	**Hauls 0 catches**		23				44	26	41		42	46	48	88	284	106	333	23	178	103	62
	**Hauls with catches**		0				0	0	0		0	0	0	1	0	0	10	0	25	14	2
	**Bycatch frequency**		0				0	0	0		0	0	0	0.01	0	0	0.03	0	0.12	0.12	0.03
	**Individuals caught**		0				0	0	0		0	0	0	1	0	0	12	0	45	21	2
	**Mean CPUE**		0				0	0	0		0	0	0	0.009	0	0	0.04	0	0.22	0.17	0.03
***d) Pteroplatytrigon violacea***																					
**Year**		**1** **(42.7–43.4 91 m)**				**2** **(43.5–44.0 71 m)**				**3** **(44.1–44.6 58 m)**				**4** **(44.7–45.2 31)**				**5** **(45.3–45.8 39)**			
		**Q1**	**Q2**	**Q3**	**Q4**	**Q1**	**Q2**	**Q3**	**Q4**	**Q1**	**Q2**	**Q3**	**Q4**	**Q1**	**Q2**	**Q3**	**Q4**	**Q1**	**Q2**	**Q3**	**Q4**
**2006**	**Hauls 0 catches**				31				46							73	119			221	167
	**Hauls with catches**				0				0							1	9			12	6
	**Bycatch frequency**				0				0							0.01	0.07			0.05	0.03
	**Individuals caught**				0				0							1	12			13	7
	**Mean CPUE**				0				0							0.01	0.09			0.06	0.03
**2007**	**Hauls 0 catches**					51								88		114	358				135
	**Hauls with catches**					0								0		5	20				6
	**Bycatch frequency**					0								0		0.04	0.05				0.04
	**Individuals caught**					0								0		8	29				6
	**Mean CPUE**					0								0		0.06	0.07				0.04
**2008**	**Hauls 0 catches**		56			56	31		41	51				359	289	147	74	23	216	170	117
	**Hauls with catches**		3			0	0		0	0				0	9	9	2	0	3	13	3
	**Bycatch frequency**		0.05			0	0		0	0				0	0.03	0.06	0.03	0	0.01	0.07	0.02
	**Individuals caught**		3			0	0		0	0				0	9	11	7	0	3	15	3
	**Mean CPUE**		0.04			0	0		0	0				0	0.03	0.08	0.11	0	0.02	0.08	0.02
**2009**	**Hauls 0 catches**				37				25			34	30	29	42	151	238				
	**Hauls with catches**				0				0			1	0	0	0	10	18				
	**Bycatch frequency**				0				0			0.03	0	0	0	0.06	0.07				
	**Individuals caught**				0				0			1	0	0	0	12	35				
	**Mean CPUE**				0				0			0.03	0	0	0	0.08	0.14				
**2010**	**Hauls 0 catches**	56	37		25	56	68		75	90	58		125	309	262	205	188	73	201	114	58
	**Hauls with catches**	0	0		0	0	0		1	0	0		2	0	8	1	15	0	7	11	7
	**Bycatch frequency**	0	0		0	0	0		0.01	0	0		0.02	0	0.03	0.005	0.07	0	0.03	0.09	0.11
	**Individuals caught**	0	0		0	0	0		1	0	0		2	0	12	1	16	0	9	26	10
	**Mean CPUE**	0	0		0	0	0		0.01	0	0		0.02	0	0.05	0.008	0.08	0	0.04	0.18	0.13
**2011**	**Hauls 0 catches**		25	30	26			37	160				112	102	90	66	442	33	271	62	46
	**Hauls with catches**		0	0	0			1	0				3	0	3	4	44	1	1	8	0
	**Bycatch frequency**		0	0	0			0.02	0				0.03	0	0.03	0.06	0.09	0.03	0.003	0.11	0
	**Individuals caught**		0	0	0			1	0				3	0	5	5	62	1	2	8	0
	**Mean CPUE**		0	0	0			0.02	0				0.04	0	0.06	0.10	0.13	0.03	0.007	0.12	0
**2012**	**Hauls 0 catches**		48			83	102			98	92		23	264	212	35	360	0	229	132	135
	**Hauls with catches**		1			0	0			0	0		3	2	12	4	46	0	2	6	8
	**Bycatch frequency**		0.02			0	0			0	0		0.11	0.007	0.05	0.10	0.11	0	0.008	0.04	0.05
	**Individuals caught**		1			0	0			0	0		3	2	12	5	57	0	2	7	9
	**Mean CPUE**		0.02			0	0			0	0		0.17	0.005	0.05	0.13	0.15	0	0.009	0.04	0.07
**2013**	**Hauls 0 catches**						28	47		80	85	33		287	244	26	120	24	317	107	77
	**Hauls with catches**						0	8		0	0	1		2	7	1	12	0	4	5	3
	**Bycatch frequency**						0	0.14		0	0	0.03		0.007	0.03	0.04	0.09	0	0.012	0.04	0.04
	**Individuals caught**						0	9		0	0	1		2	8	2	24	0	4	7	3
	**Mean CPUE**						0	0.20		0	0	0.04		0.007	0.03	0.06	0.18	0	0.012	0.06	0.03
**2014**	**Hauls 0 catches**	30				27								153	58						
	**Hauls with catches**	0				0								3	1						
	**Bycatch frequency**	0				0								0.02	0.02						
	**Individuals caught**	0				0								3	1						
	**Mean CPUE**	0				0								0.02	0.02						
**2015**	**Hauls 0 catches**		23				43	21	40		39	44	47	89	280	104	326	23	199	113	64
	**Hauls with catches**		0				1	5	1		3	2	1	0	4	2	17	0	4	4	0
	**Bycatch frequency**		0				0.02	0.19	0.02		0.07	0.04	0.02	0	0.01	0.02	0.05	0	0.02	0.02	0
	**Individuals caught**		0				1	6	1		4	3	1	0	5	2	23	0	4	4	0
	**Mean CPUE**		0				0.04	0.2	0.02		0.11	0.0	0.02	0	0.02	0.02	0.07	0	0.02	0.03	0

### CPUE standardization models

For all four species, the excess of zeros was assessed empirically from the summary of catch data (Table 2); the dispersion test confirmed the overdispersion of catch data (S2 Table).

The sets of explanatory variables included in the ZINB model computed for each species are reported in Table 3. The relative importance of each covariate in each model was assessed in terms of AIC reduction. The bigger was the reduction, the larger was the importance of a covariate for each species. The explanatory variables considered in in the backward selection procedure differed among species (see Table 3). The common smooth-hound model included five explanatory variables in the count component and three explanatory variables in the zero component (see Table 3a). The spiny dogfish model include five explanatory variables in the count component and four explanatory variables in the zero component (see Table 3b). The common eagle ray model included three explanatory variables in the count component and five explanatory variables in the zero component (see Table 3c). The pelagic stingray included four explanatory variables in the count component and three explanatory variables in the zero component (see Table 3d). The annual effect yielded large AIC reduction for common smooth-hound and common eagle ray in the count component (see Table 3a and 3c). Depth effect yielded large AIC reduction for spiny dogfish, common eagle ray and pelagic stingray in the zero component (see Table 3b, 3c and 3d). Regional effect yielded large AIC reduction for common eagle ray in the zero model (see Table 3a and 3c) and for pelagic stingray in the count component (see Table 3d).

**Table 3:** Summary statistics of the explanatory variables of ZINB models of the four elasmobranch species: a) *Mustelus mustelu*s, b) *Squalus acanthias*; c) *Myliobatis aquila* and d) *Pteroplatytrigon violacea*. The table reports Chi^2^ of likelihood ratio test, degree of freedom, *p-value* of the test and ΔAIC. Thick line indicates that dropping the variable, the model does not converge.

a) *Mustelus mustelus*					c) *Myliobatis aquila*				
	**Chi**	***df***	***p***	**ΔAIC**		**Chi**	***df***	***p***	**ΔAIC**
**Count Model**					**Count Model**				
Depth	19.08	22	<0.01	17.08	Quarter	25.58	26	<0.01	19.58
Nursery	29.84	22	<0.01	27.84	Year	162.11	21	<0.01	146.11
Quarter	43.55	20	<0.01	37.55	SST	-	-	-	-
SST	22.21	22	<0.01	20.21					
Year	76.91	14	<0.01	58.91					
**Zero Model**					**Zero Model**				
Region	45.46	20	<0.01	39.46	Region	228.33	28	<0.01	226.33
Depth	37.36	22	<0.01	35.36	Depth	100.12	28	<0.01	98.12
Mating	39.90	22	<0.01	37.90	Quarter	32.10	26	<0.01	26.10
					SST	9.78	28	<0.01	7.78
					Year	53.72	21	<0.01	37.72
b) *Squalus acanthias*					d) *Pteroplatytrygon violacea*				
	**Chi**	***df***	***p***	**ΔAIC**		**Chi**	***df***	***p***	**ΔAIC**
**Count Model**					**Count Model**				
Depth	30.94	31	<0.01	28.94	Region	95.02	18	<0.01	89.02
Nursery	76.45	31	<0.01	74.45	Depth	73.59	20	<0.01	71.59
Quarter	47.80	29	<0.01	41.80	Quarter	104.03	19	<0.01	100.03
SST	38.65	31	<0.01	36.65	SST	23.37	20	<0.01	21.37
Year	41.69	23	<0.01	23.69					
**Zero Model**					**Zero Model**				
Region	46.67	29	<0.01	40.67	Depth	106.96	20	<0.01	104.96
Depth	225.29	31	<0.01	223.29	SST	89.62	20	<0.01	87.62
SST	20.24	31	<0.01	18.24	Year	45.77	12	<0.01	27.77
Year	64.02	23	<0.01	46.02					

The nursery area and mating behaviour effect were tested only for common smooth-hound and spiny dogfish (see S1 and S2 Figs). The nursery area involved the largest AIC reduction in spiny dogfish (Table 3b), whereas it was substantially lower in common smooth-hound (Table 3a). In the common smooth-hound model, the mating behaviour effect generated a moderate AIC reduction (Table 3a) compared with the other covariates. Basing on the variable selection criteria, the effect of mating behaviour for spiny dogfish was not included in the model.

### Trends of standardized CPUE and predicted bycatch

**Fig 2:**
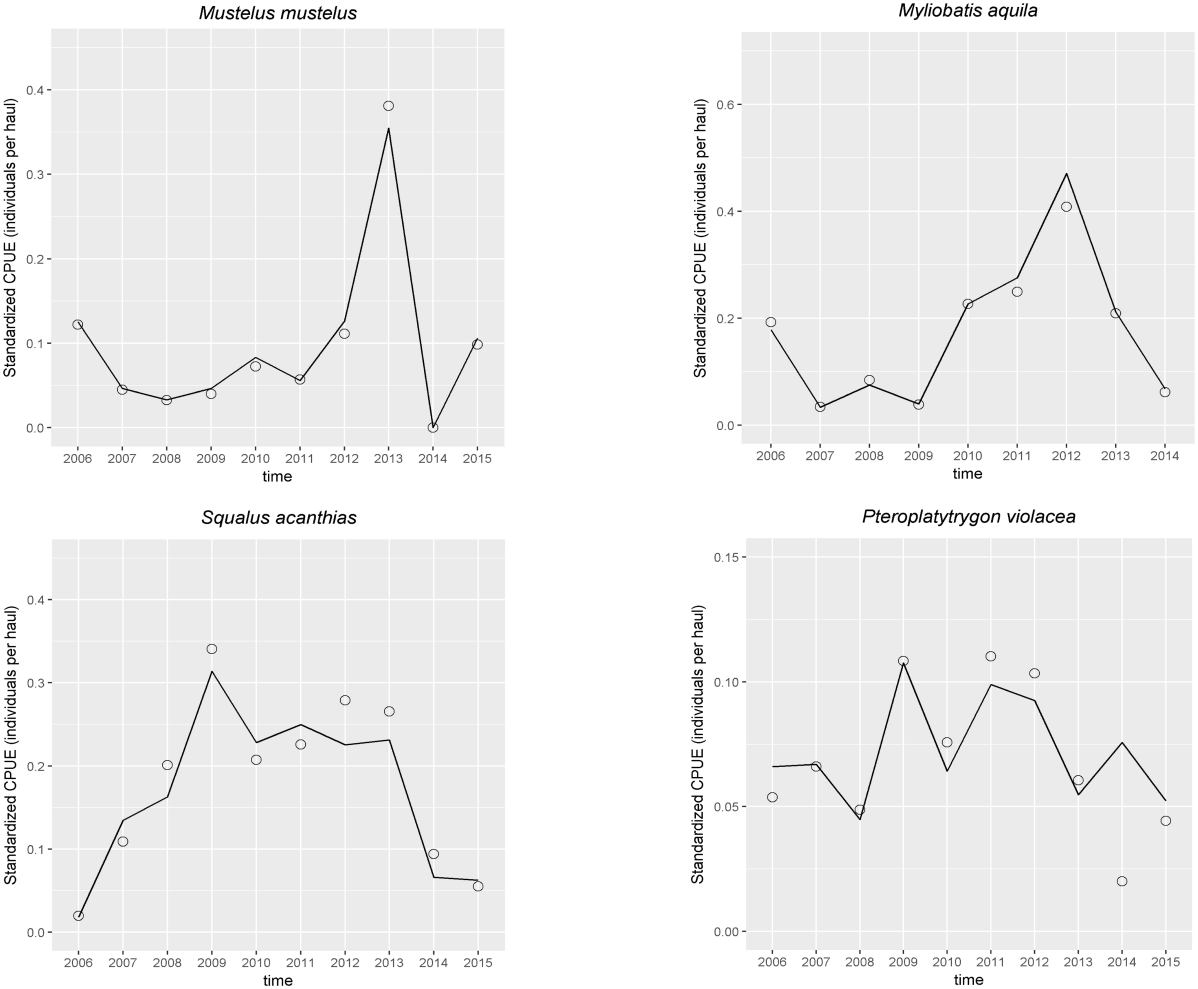
Results of CPUE standardization of *Mustelus mustelus* (a), *Squalus acanthias*, (b), *Myliobatis aquila* (c), and *Pteroplatytrygon violacea* (d). Empty dots represent annual nominal CPUE and solid lines represent annual standardized CPUE.

The nominal mean CPUE, the annual mean standardized CPUE are reported in Fig 2. The estimated time trend coefficients were not statistically significant for all species.

**Fig 3:**
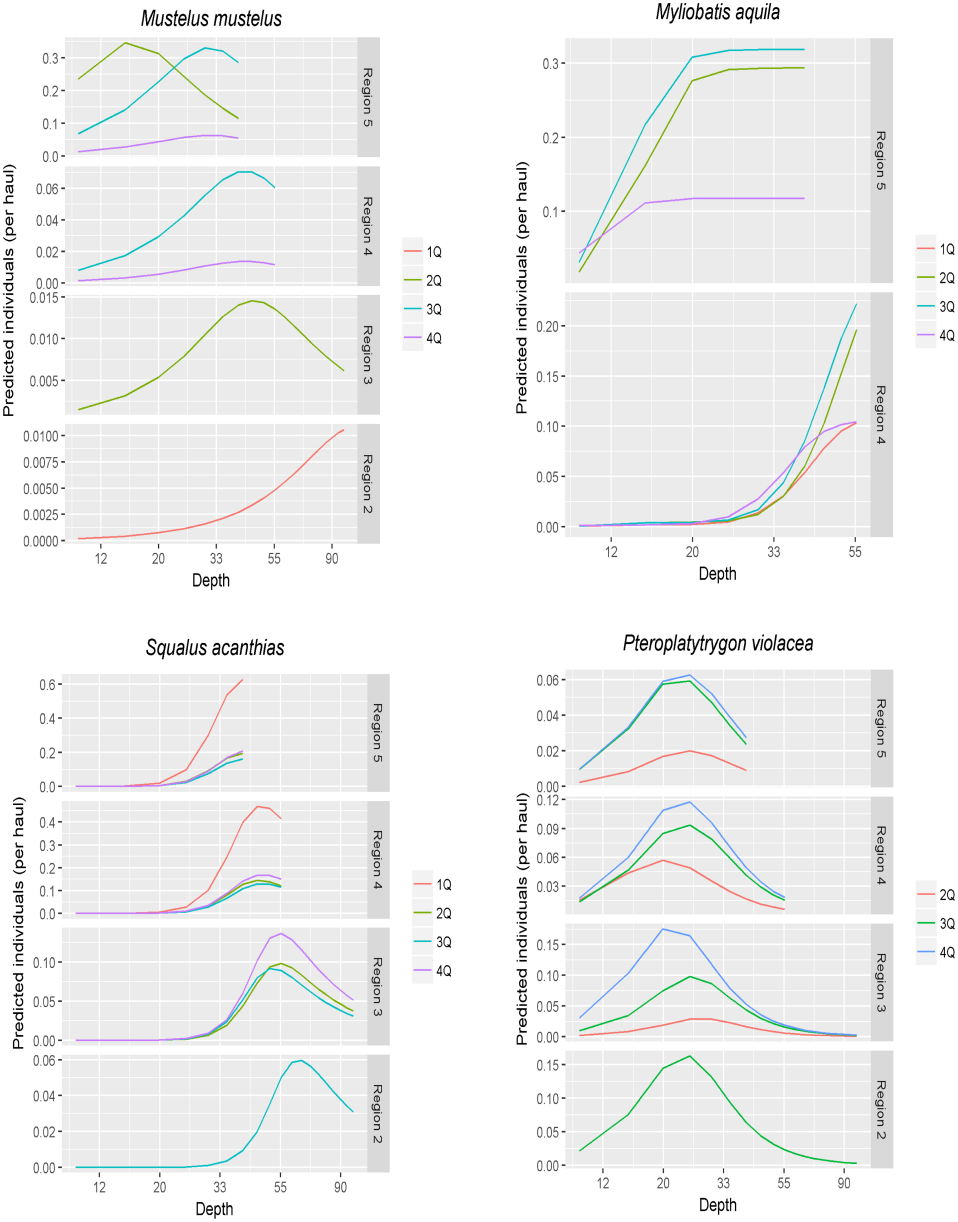
Predicted catch rate versus depth divided by regions (2–5) and quarters (1–4). For each species, only regions and quarters used in the CPUE standardization are reported.

Mean catch rates were predicted by region and quarter using the overall mean of SST and depth gradient of each region (Fig 3). The prediction of the number of individuals caught in each haul indicated seasonal variability. The depth at which catch maxima were predicted varied among fishing regions for all species except pelagic stingray, where it was stable.

## Discussion

Incidental capture during commercial fishing operations poses a major threat to long-lived marine species of conservation concern worldwide. The present study is among the first to provide an evaluation of the bycatch phenomena of different elasmobranch species in relation to pelagic/midwater trawling in the northern central Adriatic Sea. Bycatch events were concentrated in the northern Adriatic Sea, in the fishing areas above latitude 43.1, which are characterized by high fishing pressure and latitude-related biotic factors. Indeed, the northern Adriatic Sea is the most heavily exploited area in the Mediterranean, and bycatch related to long-standing, intense exploitation has been suggested to be a major reason for the continuous decline of elasmobranch species [18, 19, 41]. Furthermore, the northern Adriatic Sea is strongly influenced by the interannual freshwater discharge of the Po river, which is the chief source of nutrients in the whole basin [42, 43] and makes this area one of the most productive in the Mediterranean. Since the northern Adriatic seems to be an important nursery and reproductive habitat for many elasmobranchs [44–47], identifying critical habitats of vulnerable species like sharks and rays is of paramount importance to design ad hoc management and conservation strategies.

The 10-year data examined in this study demonstrate that the species most commonly involved in bycatch events were relatively small demersal ones like common smooth-hound (*M*. *mustelus*), spiny dogfish (*S*. *acanthias*), and common eagle ray (*M*. *aquila*). In the Adriatic Sea, their interactions with fishing gears have usually been ascribed to bottom trawlers [48, 49], trammel nets [50] and, recently, pelagic/midwater trawlers [21–23, 51]. The present data show that demersal elasmobranchs were also caught by pelagic/midwater trawlers in the northern Adriatic Sea, which is characterized by relatively shallow waters (< 50 m). Since in the Adriatic Sea demersal elasmobranchs live on soft, sandy and muddy-sandy bottoms of the continental shelf up to a depth of 200 m [52], the pelagic trawls that caught these individuals must have been towed close to the bottom.

Analysis of the present dataset indicates that the majority of common smooth-hound (*M*. *mustelus*) were caught incidentally between April and June in the northern fishing area (fishing region #5) at a depth of 15–36 m and were relatively small individuals (ca. 90 cm). The model predicted that more individuals would be caught in spring and summer in relatively shallow waters (15–30 m) in the northern Adriatic and that values would be ten times lower in late winter and spring in deeper waters (> 55 m) in the central Adriatic. Seasonal migration has recently been documented in this species [52], whose distribution area in summer seems to be limited to the northern Adriatic, whereas in autumn it extends to the central Adriatic [52]. The mating season of common smooth-hound seems to be in May [36], and pregnant females and juveniles have been found in the northern Adriatic [53] However, further information on size at maturity, fertility, and seasonal reproduction patterns is clearly needed. The present findings analysis also show that the standardized bycatch rate of common smooth-hound gradually increased from 2009 and peaked in 2013. Evidence of increasing bycatch trends has already been provided by standardized data collected during scientific surveys conducted in the Adriatic Sea between 1963 and 2005 [18], and a similar trend has recently been computed based on landing data recorded at the fish market in Chioggia (northern Adriatic) between 1997 and 2011 [53]. A large number of juveniles are usually sold at Chioggia harbour, which hosts the largest fishing fleet in the Adriatic [19, 54]. Even though common smooth-hound is a seasonal target species of trammel nets, a large number of immature individuals are landed as bycatch of pelagic/midwater and bottom trawlers in the northern Adriatic [54]. The present findings indicate that the nursery area of this species is in the northern Adriatic Sea, and aggregations of immature individuals in this area resulted in a greater mean CPUE compared with other areas. These data are in accordance with previous investigations, suggesting that the northern Adriatic should be recognized as an important nursery area for common smooth-hound and that its implications for fisheries management should carefully be considered.

A large number of spiny dogfish (*S*. *acanthias*) were incidentally captured between July and September in the central-northern area (region #4) at a depth of 30–55 m. However, according to the model, bycatch events should be most numerous in winter here and in the northern area (region #5) in relatively shallow waters (from 30 to 55 m), and lower in summer and autumn in the central Adriatic (regions #2 and #3) in deeper waters (55–90 m). Spiny dogfish is a highly vagile species, whose reported long- and short-distance seasonal migrations are generally related to feeding behaviour [55, 56]. Moreover, the data showed that most specimens were small individuals (ca. 70 cm) and were sometimes caught as aggregations of 5 to 146 individuals. As in the case of common smooth-hound, the Chioggia fish market dataset recorded the landing of a large number of immature spiny dogfish individuals [53]. In addition, it has been documented that in the North Atlantic spiny dogfish, including small individuals, often form schools segregated by size and sex [56, 57]. A similar behaviour may also occur in the Adriatic. As in the case of common smooth-hound, the aggregation of immature individuals resulted in a higher mean CPUE in the nursery area. However, since the area identified as spiny dogfish nursery area spans the northern and central-northern regions, it is possible that the effect of aggregation on mean CPUE was masked by the region effect. Despite some clear fluctuations, the model showed a relatively stable trend of standardized catches between 2006 and 2015. This pattern is consistent with the landing data from the Chioggia fish market [53]. Most spiny dogfish and common smooth-hound incidentally captured by pelagic trawlers were retained on board and sold. Both are valuable commercial species in the Adriatic Sea [17]. The life history traits of elasmobranchs make these species highly vulnerable to overexploitation [58, 59], and a significant decline in their biomass has been recorded over the past few decades [18, 19]. Common smooth-hound and spiny dogfish are currently assessed as Endangered and Critically Endangered species, respectively, in the Red List of Italian Vertebrates [60, 61]. Management measures (e.g. fishing closure in critical habitats) are therefore urgently required to restore their stocks in the Adriatic.

According to the dataset, most common eagle ray (*M*. *aquila*) individuals were incidentally caught between April and September in the northern Adriatic area (regions #4 and 5) at 20–40 m. These findings are consistent with the model. A temporal distribution pattern has recently been reported for common eagle ray in the Adriatic Sea [52]. Furthermore, the standardized catches of this species increased from 2009 to 2015, consistent with temporal patterns that have recently been documented in this area [51] and with a significant increase in standardized catches that has been calculated based on scientific surveys [18]. Since common eagle ray is usually discarded alive at sea, due to its low commercial value, it is reasonable to hypothesize that survival rate post-capture is high [21]. Yet populations seem to be declining in the Mediterranean [62, 63], where the species is currently assessed as Vulnerable by the IUCN Red List due to high bycatch rates by a variety of commercial fishing gears [64]. However, since very little is known on common eagle ray biology, further work is needed to understand the real impact of incidental capture on this species and its ecological and management implications in the Adriatic Sea.

The shallow bottoms of the Adriatic Sea entail that demersal elasmobranchs are exposed to a variety of fishing gears, including pelagic trawls. However, a considerable number of pelagic species, like pelagic stingray (*P*. *violacea*) individuals were caught in this area. The vast majority were caught between July and September in the central-northern area (region #4) at 10–35 m. A similar pattern has been described in the Gulf of Trieste (North-eastern Adriatic), where most specimens were caught in summer [65]. However, based on the modelling prediction, numbers should be relatively higher in autumn in the northern-central area (region #4) at approximately 25 m. Very little is known about the biology of this species and its occurrence in the Adriatic Sea. Nevertheless, the present findings are consistent with previous evidence of the presence of pelagic stingray in the northern Adriatic Sea [65–68]. Its scarce commercial value entails that over the past few decades it has usually been discarded at sea [21–23, 66]. The IUCN has assessed pelagic stingray as a Least Concern species in the Mediterranean [69]. Notably, it is also a frequent bycatch species in pelagic longline fisheries targeting tuna and billfish worldwide [70]. Analysis of the present dataset showed that the standardized bycatch rate of pelagic stingray decreased from 2009 to 2014, although the magnitude of the total catch is unknown and could have important implications for the population. Thus, further research is required to evaluate the population structure and monitor incidental catches of this species.

Analysis of the 10-year dataset identified a number of ecological factors as possible drivers of interactions between four elasmobranch species and pelagic/midwater trawls in the Adriatic Sea. The study considered a small range of variables. Water depth, season, and fishing regions strongly influenced the bycatch of all four species. A nursery area, identified in the northern Adriatic, was probably a major factor affecting the bycatch of common smooth-hound and spiny dogfish.

The study suffers a number of constrains. First, like common smooth-hound and spiny dog fish, common eagle ray may also aggregate in small schools [71] and this behaviour is recorded in the present dataset. However, the very limited evidence about the size at sexual maturity and mating time of this species precluded testing the mating and nursery effects. Second, the abundance and distribution of small pelagic fish are likely to provide further insight into the interaction between cartilaginous fish and pelagic trawl fisheries in the Adriatic Sea. Anchovy and sardine are the main target species of pelagic/midwater trawl fisheries in the Adriatic, and their exploitation [72, 73] may have important implications for some elasmobranchs that partially rely on them. Third, the large number of zero observations may have biased the prediction of the mean number of individual caught for each haul. The absence of bycatch data in some seasons and fishing regions may be due to a low probability to encounter elasmobranchs (non-target) during pelagic/midwater trawling, to a non- equal distribution of those species and monitoring activities in the fishing regions explored in this study and to the lack of bycatch data from the eastern Adriatic Sea. Over the last decades, spatio-temporal changes in the elasmobranch community have been detected in different scientific surveys carried out in the Adriatic Sea [18, 74]. Those changes may be influenced by a number of factors such as the peculiar environmental features of the Adriatic Sea, species life-history traits and different spatial gradients of historical fishing activities operating along both sides of the Adriatic Sea [18]. Notably, in the eastern Adriatic Sea, elasmobranchs are apparently higher in terms of abundance and diversity due to less fishing pressure compared to western Adriatic Sea [18]. In addition, some cartilaginous fish might migrate from west to east and from north to south Adriatic [52], a hypothesis that would be consistent with the present modelling prediction of a tenfold higher mean CPUE of all four species in northern and northern-central regions compared with southern regions.

Knowledge of the biology and ecology of some elasmobranch species, especially common eagle ray and pelagic sting ray, is still scanty and research directed at reducing their bycatch is still limited. Although technological mitigation measures have recently been tested in the Mediterranean [75–77], the investigation of technical innovations in this area is lagging behind. A better understanding of the real impact and ecological implications of incidental elasmobranch captures in pelagic trawl fisheries is needed, as are appropriate management measures.

## Conclusions

Evaluation of elasmobranch bycatch in commercial fisheries is of paramount importance to establish management and conservation measures for vulnerable species. Currently, very few species-specific measures are in place in the Mediterranean Sea (e.g. full ban on catching, storing, landing, trans-shipping, and selling the species covered by the Barcelona Convention and Recommendation GFCM/36/2012/3 [78]), and monitoring programmes assessing the impact of bycatch of cartilaginous fish are in their early stages in this area. The present study provides comprehensive bycatch data of four different elasmobranch species in a major Mediterranean basin. The information collected by observers on board Italian pelagic/midwater trawlers during an extensive monitoring programme offers a unique opportunity to document bycatch events of a number of species of conservation concern in the Adriatic Sea. The modelling approach used in the study fitted the empirical patterns of observed bycatch events and overcame the problem posed by an excess of zero observations, proving to be a useful strategy that can be applied to assess bycatch related to other fishing gears and other fishing areas.

## Supporting information

S1 Table Summary table reporting the total number of *M*. *mustelus* (a) and *S*. *acanthias* (b) bycaught during the monitoring programme conducted between 2006 and 2015. The total number of immature individuals, their mean length and standard deviations are listed for all five fishing regions (1–5) and calendar quarters (1Q-4Q).
(DOCX)

S2 Table Results of the dispersion test performed with full Poisson model for all species. (DOCX)

S1 Fig Differences in mean levels of estimated CPUE of *M*. *mustelus* (on the left) and *S*.*acanthias* (on the right) among fishing regions (1–5) during mating period and other periods. On the Y axis, each fishing regions (1–5) is represented on the left of the x, while the reproductive period effect is the number on the right and it has two levels (1 = mating period, 0 = other period). Significant differences in mean level of catch rate are highlighted in red.
(JPEG)

S2 Fig Differences in mean levels of CPUE of *M*. *mustelus* (on the left) and *S*.*acanthias* (on the right) within potential nursery areas and fishing area (#1-#5). (JPEG)
