# Supplementary material for: Elasmobranch bycatch in the Italian Adriatic pelagic trawl fishery
Source: PLoS One. 2018 Jan 29;13(1):e0191647. doi: 10.1371/journal.pone.0191647 (PMC5788366; doi:10.1371/journal.pone.0191647)
Supplement: S1 Table — The total number of immature individuals, their mean length and standard deviations are listed for all five fishing regions (1–5) and calendar quarters (1Q-4Q). (DOCX) [file pone.0191647.s001.docx]

**S1Table.** Summary table reporting the total number of *M. mustelus* (a) and *S. acanthias* (b) bycaught during the monitoring programme conducted between 2006 and 2015. The total number of immature individuals, their mean length and standard deviations are listed for all five fishing regions (1-5) and calendar quarters (1Q-4Q).

| 1. ***Mustelus mustelus*** | | | | | | | | | | | | | | | | | | | | | |
| --- | --- | --- | --- | --- | --- | --- | --- | --- | --- | --- | --- | --- | --- | --- | --- | --- | --- | --- | --- | --- | --- |
| **Year** | | **1**  **(42.7-43.4 91 m)** | | | | **2**  **(43.5-44.0 71 m)** | | | | **3**  **(44.1-44.6 58 m)** | | | | **4**  **(44.7-45.2 31 m)** | | | | **5**  **(45.3-45.8 39 m)** | | | |
|  |  | **Q1** | **Q2** | **Q3** | **Q4** | **Q1** | **Q2** | **Q3** | **Q4** | **Q1** | **Q2** | **Q3** | **Q4** | **Q1** | **Q2** | **Q3** | **Q4** | **Q1** | **Q2** | **Q3** | **Q4** |
| **2006** | **N individuals caught** |  |  |  |  |  |  |  |  |  |  |  |  |  |  |  | 5 |  |  | 54 | 11 |
|  | **N immature individuals** |  |  |  |  |  |  |  |  |  |  |  |  |  |  |  | 1 |  |  |  | 4 |
|  | **Mean length** |  |  |  |  |  |  |  |  |  |  |  |  |  |  |  | 35 |  |  |  | 64.5 |
|  | **Standard deviation** |  |  |  |  |  |  |  |  |  |  |  |  |  |  |  |  |  |  |  | 24.45 |
| **2007** | **N individuals caught** |  |  |  |  |  |  |  |  |  |  |  |  |  |  | 2 | 18 |  |  |  | 8 |
|  | **N immature individuals** |  |  |  |  |  |  |  |  |  |  |  |  |  |  | 2 | 17 |  |  |  | 8 |
|  | **Mean length** |  |  |  |  |  |  |  |  |  |  |  |  |  |  | 77.5 | 72.12 |  |  |  | 66.12 |
|  | **Standard deviation** |  |  |  |  |  |  |  |  |  |  |  |  |  |  | 24.75 | 13.17 |  |  |  | 20.40 |
| **2008** | **N individuals caught** |  |  |  |  |  |  |  |  |  |  |  |  |  |  |  | 1 |  | 12 | 14 | 1 |
|  | **N immature individuals** |  |  |  |  |  |  |  |  |  |  |  |  |  |  |  |  |  | 6 | 11 | 1 |
|  | **Mean length** |  |  |  |  |  |  |  |  |  |  |  |  |  |  |  |  |  | 85.83 | 68.91 | 105 |
|  | **Standard deviation** |  |  |  |  |  |  |  |  |  |  |  |  |  |  |  |  |  | 24.37 | 28.97 |  |
| **2009** | **N individuals caught** |  |  |  |  |  |  |  |  |  |  |  |  |  |  | 13 | 5 |  |  |  |  |
|  | **N immature individuals** |  |  |  |  |  |  |  |  |  |  |  |  |  |  | 11 | 5 |  |  |  |  |
|  | **Mean length** |  |  |  |  |  |  |  |  |  |  |  |  |  |  | 73.36 | 64.4 |  |  |  |  |
|  | **Standard deviation** |  |  |  |  |  |  |  |  |  |  |  |  |  |  | 9.76 | 20.27 |  |  |  |  |
| **2010** | **N individuals caught** |  |  |  |  | 1 |  |  |  |  |  |  |  |  | 6 | 6 |  | 55 |  | 1 |  |
|  | **N immature individuals** |  |  |  |  | 1 |  |  |  |  |  |  |  |  | 6 | 6 |  | 36 |  | 1 |  |
|  | **Mean length** |  |  |  |  | 65 |  |  |  |  |  |  |  |  | 91.5 | 56.17 |  | 85.22 |  | 80 |  |
|  | **Standard deviation** |  |  |  |  |  |  |  |  |  |  |  |  |  | 10.54 | 20.48 |  | 19.11 |  |  |  |
| **2011** | **N individuals caught** |  |  |  |  |  |  |  |  |  |  |  |  |  |  |  | 5 |  | 47 | 6 |  |
|  | **N immature individuals** |  |  |  |  |  |  |  |  |  |  |  |  |  |  |  | 5 |  | 30 | 6 |  |
|  | **Mean length** |  |  |  |  |  |  |  |  |  |  |  |  |  |  |  | 68 |  | 94.93 | 96.5 |  |
|  | **Standard deviation** |  |  |  |  |  |  |  |  |  |  |  |  |  |  |  | 7.38 |  | 17.47 | 11.57 |  |
| **2012** | **N individuals caught** |  |  |  |  | 14 |  |  |  |  | 4 |  |  |  |  | 1 | 9 |  | 72 | 26 | 16 |
|  | **N immature individuals** |  |  |  |  | 13 |  |  |  |  | 4 |  |  |  |  |  | 8 |  | 39 | 18 | 7 |
|  | **Mean length** |  |  |  |  | 79.77 |  |  |  |  | 70.5 |  |  |  |  |  | 57.75 |  | 96.05 | 90 | 62.14 |
|  | **Standard deviation** |  |  |  |  | 16.13 |  |  |  |  | 19.33 |  |  |  |  |  | 17.89 |  | 14.24 | 15.84 | 12.07 |
| **2013** | **N individuals caught** |  |  |  |  |  |  |  |  |  | 1 |  |  |  |  | 1 | 18 |  | 246 | 35 | 1 |
|  | **N immature individuals** |  |  |  |  |  |  |  |  |  | 1 |  |  |  |  | 1 | 18 |  | 201 | 32 | 1 |
|  | **Mean length** |  |  |  |  |  |  |  |  |  | 62 |  |  |  |  | 105 | 67.55 |  | 93.14 | 88.41 | 60 |
|  | **Standard deviation** |  |  |  |  |  |  |  |  |  |  |  |  |  |  |  | 20.03 |  | 16.10 | 14.06 |  |
| **2014** | **N individuals caught** |  |  |  |  |  |  |  |  |  |  |  |  |  |  |  |  |  |  |  |  |
|  | **N immature individuals** |  |  |  |  |  |  |  |  |  |  |  |  |  |  |  |  |  |  |  |  |
|  | **Mean length** |  |  |  |  |  |  |  |  |  |  |  |  |  |  |  |  |  |  |  |  |
|  | **Standard deviation** |  |  |  |  |  |  |  |  |  |  |  |  |  |  |  |  |  |  |  |  |
| **2015** | **N individuals caught** |  |  |  |  |  |  |  |  |  |  |  |  |  |  |  | 11 |  | 55 | 22 | 1 |
|  | **N immature individuals** |  |  |  |  |  |  |  |  |  |  |  |  |  |  |  | 5 |  | 37 | 19 | 1 |
|  | **Mean length** |  |  |  |  |  |  |  |  |  |  |  |  |  |  |  | 70.60 |  | 80.90 | 85.16 | 96 |
|  | **Standard deviation** |  |  |  |  |  |  |  |  |  |  |  |  |  |  |  | 21.01 |  | 35.90 | 17.41 |  |
| 1. ***Squalus acanthias*** | | | | | | | | | | | | | | | | | | | | | |

| **Year** | | **1**  **(42.7-43.4 91 m)** | | | | **2**  **(43.5-44.0 71 m)** | | | | **3**  **(44.1-44.6 58 m)** | | | | **4**  **(44.7-45.2 31 m)** | | | | **5**  **(45.3-45.8 39 m)** | | | |
| --- | --- | --- | --- | --- | --- | --- | --- | --- | --- | --- | --- | --- | --- | --- | --- | --- | --- | --- | --- | --- | --- |
|  |  | **Q1** | **Q2** | **Q3** | **Q4** | **Q1** | **Q2** | **Q3** | **Q4** | **Q1** | **Q2** | **Q3** | **Q4** | **Q1** | **Q2** | **Q3** | **Q4** | **Q1** | **Q2** | **Q3** | **Q4** |
| 2006 | **N individuals caught** |  |  |  |  |  |  |  |  |  |  |  |  |  |  |  | 7 |  |  | 3 |  |
|  | **N immature individuals** |  |  |  |  |  |  |  |  |  |  |  |  |  |  |  | 4 |  |  |  |  |
|  | **Mean length** |  |  |  |  |  |  |  |  |  |  |  |  |  |  |  | 48.5 |  |  |  |  |
|  | **Standard deviation** |  |  |  |  |  |  |  |  |  |  |  |  |  |  |  | 13.53 |  |  |  |  |
| 2007 | **N individuals caught** |  |  |  |  |  |  |  |  |  |  |  |  | 15 |  | 10 | 58 |  |  |  | 1 |
|  | **N immature individuals** |  |  |  |  |  |  |  |  |  |  |  |  |  |  | 10 | 58 |  |  |  | 1 |
|  | **Mean length** |  |  |  |  |  |  |  |  |  |  |  |  |  |  | 54.80 | 74.72 |  |  |  | 37 |
|  | **Standard deviation** |  |  |  |  |  |  |  |  |  |  |  |  |  |  | 10.39 | 10.42 |  |  |  |  |
| 2008 | **N individuals caught** |  |  |  |  |  |  |  |  |  |  |  |  | 102 | 10 | 75 | 9 | 1 | 33 | 51 | 1 |
|  | **N immature individuals** |  |  |  |  |  |  |  |  |  |  |  |  | 100 | 10 | 74 | 8 | 1 | 33 | 51 |  |
|  | **Mean length** |  |  |  |  |  |  |  |  |  |  |  |  | 72.91 | 68.30 | 49.09 | 65.37 | 27 | 51.09 | 66.19 |  |
|  | **Standard deviation** |  |  |  |  |  |  |  |  |  |  |  |  | 11.99 | 9.69 | 17.98 | 15.41 |  | 22.77 | 8.30 |  |
| 2009 | **N individuals caught** |  |  |  |  |  |  |  |  |  |  | 36 |  |  |  | 48 | 106 |  |  |  |  |
|  | **N immature individuals** |  |  |  |  |  |  |  |  |  |  | 36 |  |  |  | 48 | 98 |  |  |  |  |
|  | **Mean length** |  |  |  |  |  |  |  |  |  |  | 60.63 |  |  |  | 66.06 | 59.16 |  |  |  |  |
|  | **Standard deviation** |  |  |  |  |  |  |  |  |  |  | 12.04 |  |  |  | 14.81 | 14.00 |  |  |  |  |
| 2010 | **N individuals caught** |  |  |  |  |  |  |  |  |  | 3 |  | 13 | 136 | 19 | 76 | 78 | 4 | 17 | 2 | 3 |
|  | **N immature individuals** |  |  |  |  |  |  |  |  |  | 3 |  | 11 | 135 | 19 | 76 | 77 | 4 | 16 | 2 | 3 |
|  | **Mean length** |  |  |  |  |  |  |  |  |  | 52.33 |  | 62.73 | 73.07 | 69.26 | 66.83 | 51.89 | 66.25 | 78.94 | 57 | 63.33 |
|  | **Standard deviation** |  |  |  |  |  |  |  |  |  | 27.02 |  | 16.8 | 6.90 | 11.08 | 15.07 | 17.21 | 20.79 | 11.48 | 25.45 | 5.77 |
| 2011 | **N individuals caught** |  |  |  |  |  |  | 2 |  |  |  |  | 25 | 100 | 10 | 14 | 123 | 10 | 4 |  | 5 |
|  | **N immature individuals** |  |  |  |  |  |  | 2 |  |  |  |  | 25 | 100 | 10 | 14 | 121 | 10 | 4 |  | 5 |
|  | **Mean length** |  |  |  |  |  |  | 74 |  |  |  |  | 59.20 | 66.19 | 73.40 | 74 | 58.21 | 63.50 | 67 |  | 46.4 |
|  | **Standard deviation** |  |  |  |  |  |  | 7.07 |  |  |  |  | 15.67 | 13.79 | 15.71 | 7.51 | 20.05 | 15.83 | 5.48 |  | 12.74 |
| 2012 | **N individuals caught** |  |  |  |  |  |  |  |  |  | 7 |  | 84 | 15 |  | 16 | 108 |  | 26 | 173 | 19 |
|  | **N immature individuals** |  |  |  |  |  |  |  |  |  | 5 |  | 83 | 15 |  | 16 | 98 |  | 22 | 90 | 13 |
|  | **Mean length** |  |  |  |  |  |  |  |  |  | 73.20 |  | 68.65 | 75.73 |  | 52.12 | 62.43 |  | 73.45 | 68.70 | 73.08 |
|  | **Standard deviation** |  |  |  |  |  |  |  |  |  | 6.42 |  | 12.75 | 5.20 |  | 17.86 | 16.58 |  | 19.30 | 11.52 | 15.83 |
| 2013 | **N individuals caught** |  |  |  |  |  |  | 4 |  |  |  | 5 |  | 112 | 26 |  | 48 |  | 165 | 22 | 28 |
|  | **N immature individuals** |  |  |  |  |  |  | 3 |  |  |  | 4 |  | 96 | 26 |  | 48 |  | 160 | 22 | 28 |
|  | **Mean length** |  |  |  |  |  |  | 58 |  |  |  | 82.75 |  | 74.36 | 69.70 |  | 63.25 |  | 85.50 | 51.13 | 63.78 |
|  | **Standard deviation** |  |  |  |  |  |  | 4.36 |  |  |  | 21.93 |  | 8.31 | 12.64 |  | 18.37 |  | 17.94 | 18.90 | 12.69 |
| 2014 | **N individuals caught** |  |  |  |  |  |  |  |  |  |  |  |  | 14 | 1 |  |  |  |  |  |  |
|  | **N immature individuals** |  |  |  |  |  |  |  |  |  |  |  |  | 2 | 1 |  |  |  |  |  |  |
|  | **Mean length** |  |  |  |  |  |  |  |  |  |  |  |  | 71.50 | 85 |  |  |  |  |  |  |
|  | **Standard deviation** |  |  |  |  |  |  |  |  |  |  |  |  | 6.37 |  |  |  |  |  |  |  |
| 2015 | **N individuals caught** |  |  |  |  |  |  |  |  |  |  | 1 | 3 | 7 | 22 | 2 | 14 | 1 | 18 | 2 | 7 |
|  | **N immature individuals** |  |  |  |  |  |  |  |  |  |  | 1 | 1 | 7 | 22 | 2 | 14 | 1 | 18 | 2 | 7 |
|  | **Mean length** |  |  |  |  |  |  |  |  |  |  | 53 | 29 | 73.57 | 77.54 | 70 | 64.50 | 70 | 78.11 | 70.50 | 57.57 |
|  | **Standard deviation** |  |  |  |  |  |  |  |  |  |  |  |  | 3.95 | 8.20 | 8.48 | 19.65 |  | 16.44 | 6.37 | 19.07 |
