# Supplementary material for: Elasmobranch bycatch in the Italian Adriatic pelagic trawl fishery
Source: PLoS One. 2018 Jan 29;13(1):e0191647. doi: 10.1371/journal.pone.0191647 (PMC5788366; doi:10.1371/journal.pone.0191647)
Supplement: S2 Table — (DOCX) [file pone.0191647.s002.docx]

**S2Table.** Results of the dispersion test performed with full Poisson model for all species.

| **Species** | **α** | **z** | **p-value** |
| --- | --- | --- | --- |
| *Mustelus mustelus* | 11.9 | 8.35 | <0.001 |
| *Squalus acanthias* | 40.18 | 5.54 | <0.001 |
| *Myliobatis aquila* | 72.84 | 1.95 | 0.02 |
| *Pteroplatytrygon violacea* | 8.61 | 6.14 | <0.001 |
