# Supplementary figures and images for: Elasmobranch bycatch in the Italian Adriatic pelagic trawl fishery
Source: PLoS One. 2018 Jan 29;13(1):e0191647. doi: 10.1371/journal.pone.0191647 (PMC5788366; doi:10.1371/journal.pone.0191647)

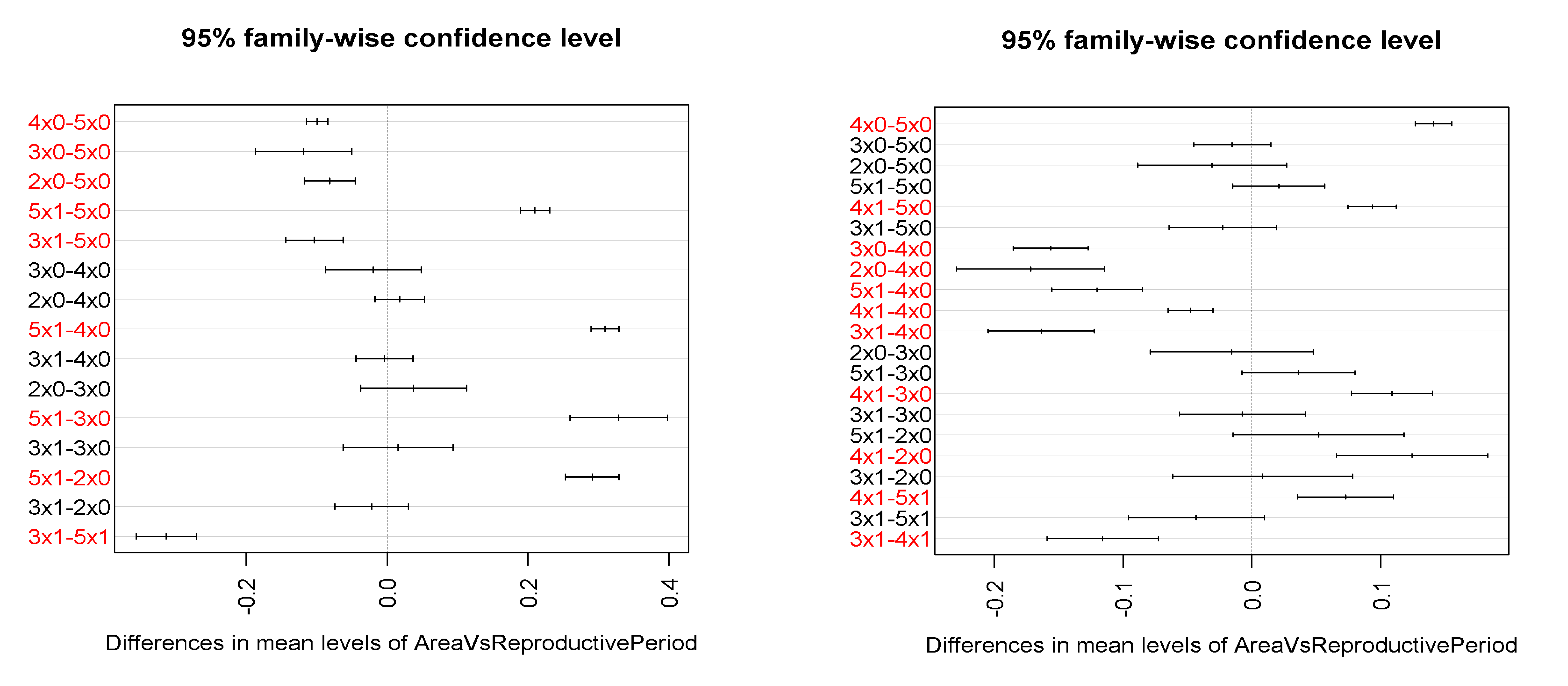

Supplement: S1 Fig — On the Y axis, each fishing regions (1–5) is represented on the left of the x, while the reproductive period effect is the number on the right and it has two levels (1 = mating period, 0 = other period). Significant differences in mean level of catch rate are highlighted in red. (JPEG) [file pone.0191647.s003.jpeg]

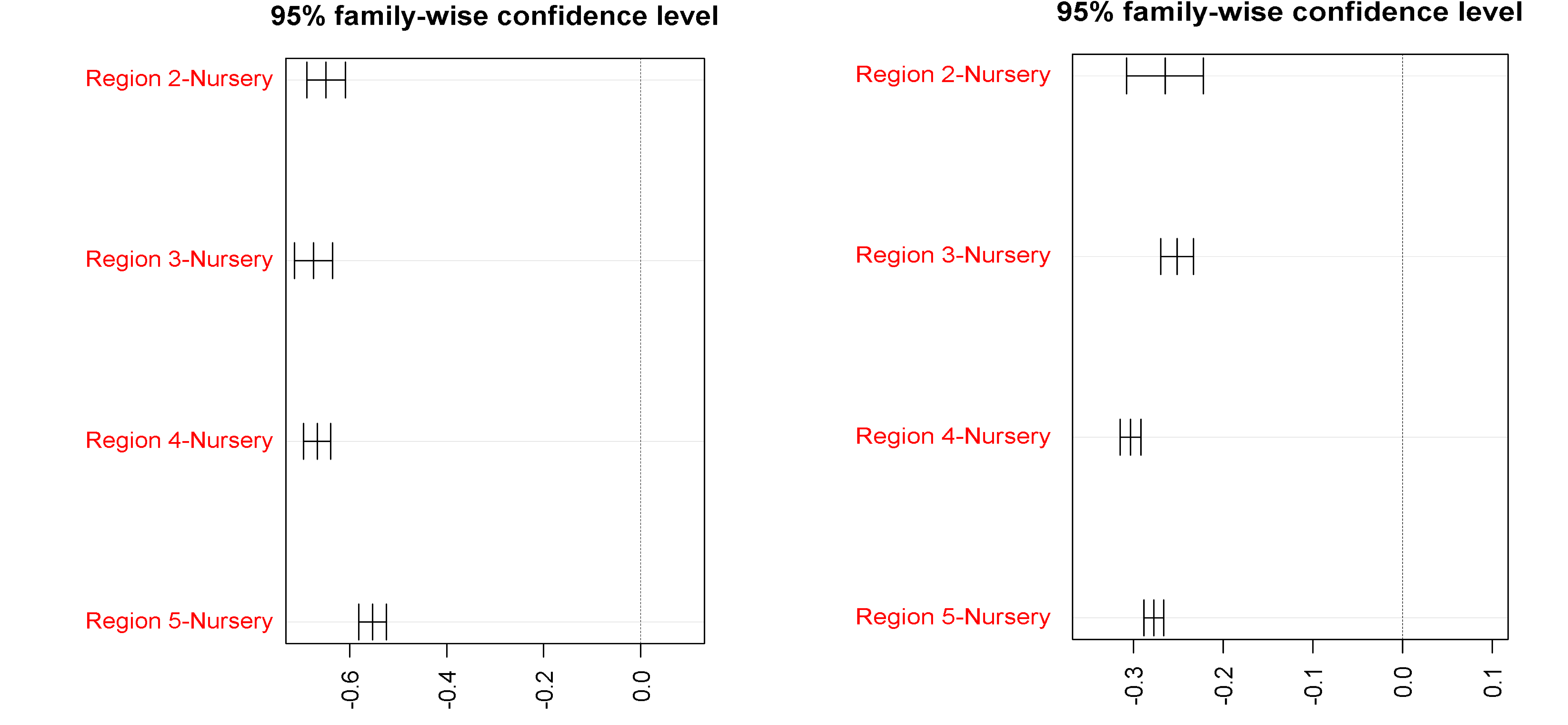

Supplement: S2 Fig — (JPEG) [file pone.0191647.s004.jpeg]
